# Preoperative prediction of Ki-67 expression and prognosis in breast cancer based on contrast-enhanced cone-beam breast CT: a two-centre study

**DOI:** 10.1186/s40644-026-01082-0

**Published:** 2026-06-26

**Authors:** Wenxiu Guo, Binglin Lyu, Yue Ma, Yafei Wang, Keyi Bian, Jiaqi Wu, Yaopan Wu, Jie Jiang, Yueqiang Zhu, Zhaoxiang Ye

**Affiliations:** 1 Department of Radiology, Medical Artificial General Intelligence for Computation (MAGIC) Lab, Tianjin Medical University Cancer Institute & Hospital; National Clinical Research Centre for Cancer; Tianjin’s Clinical Research Centre for Cancer; Key Laboratory of Cancer Prevention and Therapy, Tianjin; Key Laboratory of Breast Cancer Prevention and Therapy, Tianjin Medical University, Ministry of Education; State Key Laboratory of Druggability Evaluation and Systematic Translational Medicine, Huan-Hu-Xi Road, Ti-Yuan-Bei, Hexi District, Tianjin, 300060 China; 2 Department of Radiology, Qilu Hospital of Shandong University, Wen-Hua-Xi Road, Lixia District, Jinan, Shandong 250012 China; 3 Department of Radiology, Shandong Cancer Hospital and Institute, Shandong First Medical University and Shandong Academy of Medical Sciences, No. 440 Jiyan Road, Huaiyin District, Jinan, Shandong 250117 China; 4 Department of Medical Imaging, Jinan Third People’s Hospital, No.1 Wangsheren North Street, Industrial North Road, Licheng District, Jinan, Shandong 250132 China; 5 Department of Medical Imaging and Image-guided Therapy, Sun Yat-Sen University Cancer Centre, State Key Laboratory of Oncology in South China, Collaborative Innovation Centre for Cancer Medicine, Dong-Feng-Dong Road, Yuexiu District, Guangzhou, Guangdong 510060 China; 6 Department of Gynecology, Qilu Hospital of Shandong University, Wen-Hua-Xi Road, Lixia District, Jinan, Shandong 250012 China; 7 Department of Medical Imaging, Radboud University Medical Centre, PO Box 9101, Nijmegen, 6500 HB The Netherlands

**Keywords:** Breast neoplasms, Ki-67 Antigen, Cone-beam computed tomography, Radiomics, Deep learning

## Abstract

**Background:**

This study aimed to develop and validate preoperative contrast-enhanced cone-beam breast CT (CE-CBBCT)-based radiomics, deep learning, and combined models for predicting Ki-67 expression status and to preliminarily explore their prognostic value, with the goal of informing preoperative treatment decisions.

**Methods:**

This two-centre study enrolled 636 breast cancer patients, divided into training (*n* = 310), internal validation (*n* = 77), external test (*n* = 78), and prognostic (*n* = 171) cohorts. Two Ki-67 cut-offs (14% and 30%) were applied. Radiomics models were constructed using five machine learning classifiers, and deep learning models using four three-dimensional architectures (ResNet50, ResNet101, DenseNet121, and ShuffleNet). The best-performing models from each category were integrated with the clinical model using logistic regression to establish combined models. Prognostic value was evaluated using disease-free survival (DFS) in the prognostic cohort.

**Results:**

Among the radiomics models, those based on ExtraTrees and eXtreme Gradient Boosting performed best for the 14% and 30% cut-offs, respectively, with areas under the receiver operating characteristic curves (AUCs) of 0.885 and 0.831 in the external test cohort. Among the deep learning architectures, ShuffleNet performed best, with corresponding AUCs of 0.630 and 0.825. Combined models achieved the highest AUCs of 0.895 and 0.869 for the 14% and 30% cut-offs, respectively, in the external test cohort. However, they did not significantly outperform the corresponding radiomics models. In survival analysis, the radiomics score for predicting Ki-67 ≥ 30% was an independent prognostic factor for DFS (hazard ratio: 1.661; 95% confidence interval: 1.142–2.417; *P* = 0.008).

**Conclusions:**

CE-CBBCT-based radiomics models demonstrated utility as non-invasive tools for the preoperative prediction of high Ki-67 expression defined at both the 14% and 30% cut-offs. The prognostic value of the radiomics score for predicting Ki-67 ≥ 30% was preliminarily demonstrated.

**Supplementary Information:**

The online version contains supplementary material available at https://doi.org/10.1186/s40644-026-01082-0.

## Introduction

Breast cancer is the most common malignancy in women worldwide [1]. The Ki-67 protein, a key marker of cellular proliferation, is critical for molecular subtyping, informing therapeutic decisions, and assessing treatment response and prognosis [2–5]. High Ki-67 expression in breast cancer is generally associated with more aggressive tumour behaviour and a higher risk of recurrence; yet, it also predicts a better response to chemotherapy and an increased likelihood of achieving pathological complete response following neoadjuvant therapy [6–8]. Therefore, assessing the Ki-67 index is essential prior to selecting specific treatment strategies for patients with breast cancer.

Currently, preoperative Ki-67 evaluation relies on invasive core-needle biopsy (CNB), which is time-consuming and affected by tumour heterogeneity and inter-observer variability. Reported discordance rates between CNB and surgical specimens range from 10% to 40% [9, 10]. Given these limitations, a reliable non-invasive method that captures tumour heterogeneity is urgently needed for accurate Ki-67 assessment.

Complicating matters further, clinical practice employs varying Ki-67 cut-off values: 14% for molecular subtyping per the St. Gallen Consensus [11], and 30% for prognostic stratification and to guide chemotherapy decisions as recommended by the International Ki-67 Working Group (IKWG) [2]. However, few studies have explored Ki-67 prediction using both thresholds, limiting their clinical generalisability.

Recent studies have explored magnetic resonance imaging (MRI) and ultrasound as non-invasive tools for Ki-67 prediction [8, 12–14]. MRI provides valuable functional imaging but is limited by long scan times, high cost, and suboptimal detection of microcalcifications, which are important markers of breast cancer [15]. Conventional ultrasound, though widely accessible, real-time, and well-tolerated, is limited by operator dependency, which affects diagnostic reproducibility [16].

In this context, contrast-enhanced cone-beam breast CT (CE-CBBCT) has emerged as a promising alternative. It delivers high‑resolution three‑dimensional images with eliminated tissue superimposition. In addition, it simultaneously visualises microcalcifications and enhancement [17, 18]. These capabilities enable clearer delineation of fine tumour structures and morphological characteristics, making CE‑CBBCT an ideal platform for advanced computational approaches such as radiomics and deep learning [19–23]. Radiomics enables the extraction of quantitative features from medical images for objective lesion characterisation and precision medicine [24], while deep learning facilitates automated extraction of high dimensional patterns from complex imaging data, providing new opportunities for obtaining clinical insights [25]. The integration of these techniques with the rich morphological and functional information provided by CE-CBBCT may therefore enhance the prediction of tumour aggressiveness, particularly with respect to Ki-67 expression levels.

To our knowledge, no radiomics or deep learning studies have used CE-CBBCT to predict Ki-67 expression. Thus, this study aimed to develop and validate preoperative CE-CBBCT-based radiomics and deep learning models for predicting Ki-67 expression at two distinct cut-off values. It also evaluated their potential for preoperatively identifying patients with a poor prognosis, thereby assisting in the formulation of individualised treatment strategies.

## Methods

### Patient selection and data collection

**Fig. 1:**
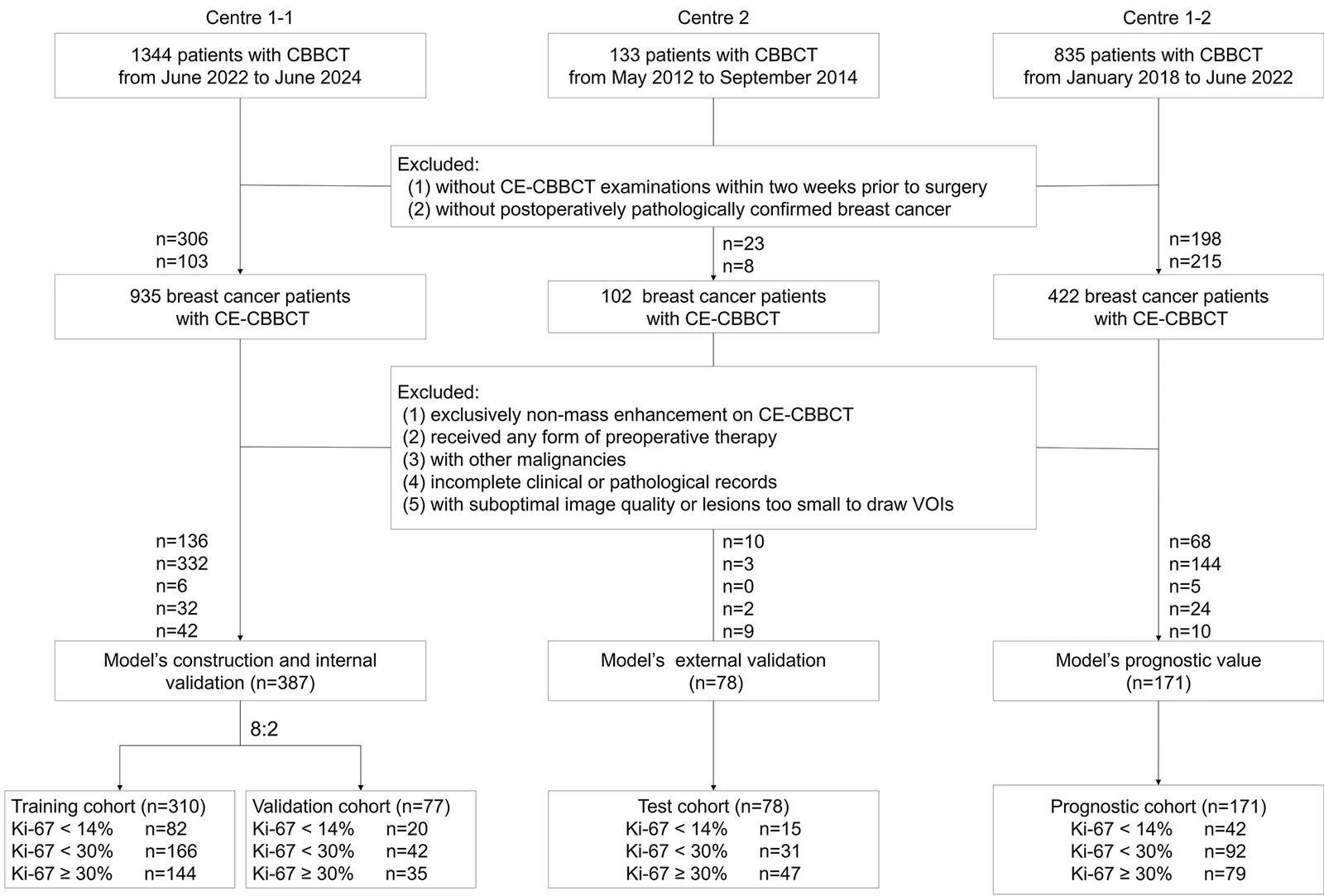
Flowchart of patient recruitment and study design. *CBBCT*, cone-beam breast CT; *CE-CBBCT*, contrast-enhanced cone-beam breast CT; *VOI*, volume of interest

The flowchart of patient selection is shown in Fig. 1. Eligible participants had pathologically confirmed breast cancer and underwent CE-CBBCT within two weeks before surgery. Exclusion criteria included: receipt of any preoperative therapy; other concurrent malignancies; incomplete clinical or pathological records (e.g., missing Ki-67 index); lesions exhibiting only non-mass enhancement (ill-defined margins or irregular shape preventing reliable segmentation); or inadequate image quality or lesion size for volume of interest (VOI) delineation. For patients with multiple or bilateral lesions, the largest lesion was selected according to established practice [26, 27].

At centre 1, 387 eligible patients (June 2022–June 2024) were included for model development and chronologically divided into training (*n* = 310; before January 2024) and internal validation (*n* = 77; after January 2024) sets at an 8:2 ratio. The external test set included 78 eligible patients from centre 2 (May 2012–September 2014). A separate prognostic validation cohort comprised 171 patients from centre 1 (January 2018–June 2022) with follow-up for survival analysis.

### CBBCT imaging protocol and preprocessing

CE-CBBCT examinations were performed at two centres using the same dedicated flat-panel detector systems (KBCT-1000, Koning Corporation) with identical standardised protocols. Patients were positioned prone with the breast suspended without compression. After a non-contrast scan, 90 mL of non-ionic contrast medium (Iohexol, Omnipaque^®^ 300, GE Healthcare) was injected intravenously at 2 mL/s. Contrast-enhanced imaging commenced at 120 s post-injection for the ipsilateral breast and about 180 s for the contralateral breast. The scanning parameters were consistent across the two centres and included a tube voltage of 49 kVp, a tube current of 50–80 mA, a scan time of 10 s, a scan volume of 16 × 28 × 28 cm, and 300 projections. Isotropic images were reconstructed with a voxel size of (0.273 mm)³. The complete bilateral examination (8–10 min) delivered an average glandular dose of 11.46 mGy per breast.

A breast radiologist independently delineated tumour VOIs on CE-CBBCT images using 3D Slicer (v4.11.20210226) while blinded to pathological outcomes. A second radiologist re-evaluated 30 randomly selected tumours to assess inter-observer consistency.

### Collection of clinicopathological information

Clinicopathological data were collected, including age, menopausal status, tumour type, histological grade, estrogen receptor (ER) status, progesterone receptor (PR) status, human epidermal growth factor receptor 2 (HER2) status, molecular subtype, lymph node (LN) status, and Ki-67 index. Ki-67 expression was assessed by immunohistochemistry (IHC) within one week of surgical resection. The Ki-67 index was defined as the percentage of positively stained nuclei among tumour cells. Based on two predefined cut-off values (14% and 30%), patients were classified into high- and low-Ki-67 expression groups. Conventional imaging data included fibroglandular tissue (FGT) category, background parenchymal enhancement (BPE), and the presence, location, focality, and size of lesions. Clinical and imaging features were evaluated for their association with Ki-67 expression, and significant predictors were used for clinical model construction.

### Development of radiomics, deep learning, and combined models

Radiomics and deep learning analyses were conducted using the Onekey AI platform. Radiomics features were extracted from both original and preprocessed images using the PyRadiomics package (version 3.0.1). Preprocessing included multiscale Laplacian-of-Gaussian filtering (σ = 1.0, 2.0, and 3.0 mm), Haar wavelet decomposition, three-dimensional (3D) local binary pattern filtering, gradient filtering, and exponential, square, square-root, and logarithmic transformations. The parameters for Z-score normalisation were estimated in the training cohort and subsequently applied unchanged to the validation and test cohorts.

The Synthetic minority oversampling technique (SMOTE) was used to address class imbalance in the training cohort. Features with an intraclass correlation coefficient (ICC) > 0.75 and a *P* value < 0.05 on either an independent *t*-test or a Mann–Whitney U test were retained. Pearson correlation analysis was then used to eliminate redundant features (|*r*| > 0.90). The 30 highest-ranked features were selected using the minimum redundancy maximum relevance (mRMR) method, after which features with non-zero coefficients were retained using least absolute shrinkage and selection operator (LASSO) regression with 10-fold cross-validation. Five machine learning classifiers, Random Forest (RF), ExtraTrees (ET), eXtreme Gradient Boosting (XGBoost), Adaptive Boosting (AdaBoost), and Multi-Layer Perceptron (MLP), were evaluated to build the radiomics model. Hyperparameters for each classifier were optimised by grid search with 5-fold cross-validation in the training cohort, using the median area under the receiver operating characteristic (ROC) curve (AUC) across folds as the selection criterion. For each classifier, the combination achieving the highest median AUC was selected, and the final model was then retrained on the entire training cohort. The search spaces and selected hyperparameters for each classifier are provided in Supplementary Table S1. The radiomics score was defined as the predicted probability of high Ki-67 expression generated by the radiomics model. SHapley Additive exPlanations (SHAP) was used to visualise the model prediction process.

We developed and compared four three-dimensional (3D) deep learning architectures for predicting high Ki-67 expression: 3D ShuffleNet, 3D ResNet50, 3D ResNet101, and 3D DenseNet121. For each lesion, a 3D VOI was generated based on the minimum bounding box of the tumour segmentation with limited peritumoural context. Each network automatically learned features from the 3D VOIs during training, eliminating the need for predefined feature extraction or selection. To enhance dataset diversity, we applied data augmentation by flipping the images along the x-, y-, and z-axes. All deep learning models were trained end-to-end under a unified training protocol to ensure fair comparison across architectures. Briefly, model optimisation was performed using stochastic gradient descent with an initial learning rate of 0.001, momentum of 0.9, and weight decay of 1 × 10^− 4^. Training was conducted for 80 epochs, and an adaptive learning rate scheduling strategy was applied. Regularisation strategies, including weight decay and data augmentation were used to mitigate overfitting. Detailed training settings are provided in Supplementary Table S2. Additional implementation details, including the programming language, software libraries, deep learning framework, and specific libraries used for SMOTE and SHAP analyses, are provided in the Supplementary Materials.

**Fig. 2:**
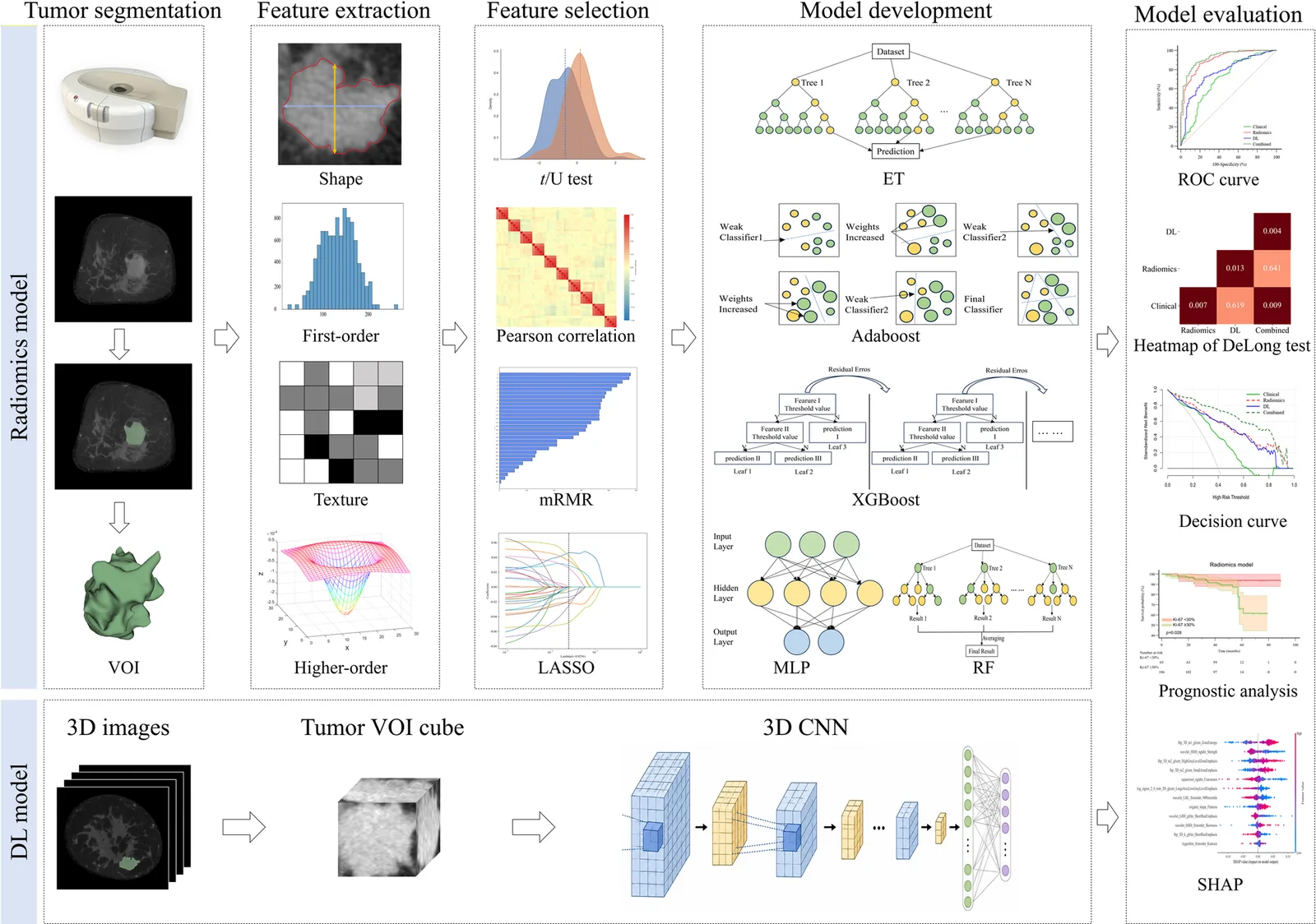
Workflow of the research procedure. *VOI*, volumes of interest; *mRMR*, minimum redundancy maximum relevance; *LASSO*, least absolute shrinkage and selection operator; *ET*, ExtraTrees; *AdaBoost*, Adaptive Boosting; *XGBoost*, eXtreme Gradient Boosting; *MLP*, Multi-Layer Perceptron; *RF*, Random forest; *ROC*, receiver operating characteristic; *SHAP*, SHapley Additive exPlanation; *3D CNN*, three-dimensional convolutional neural network

A combined model was developed using logistic regression to integrate the predicted probabilities from the best-performing radiomics, deep learning, and clinical models. The overall workflow is illustrated in Fig. 2.

### Survival analysis

Disease-free survival (DFS) was defined as the interval in months from the date of surgery to the occurrence of events such as invasive local, regional, or distant recurrences, contralateral breast cancer, second primary non-breast cancer, death from any cause, or in situ cancer events [28]. Recurrence status was determined through medical records and telephone follow-up until June 1, 2025. Patients without events or those lost to follow-up were censored. The median follow-up was 50 months (range, 3–89 months). Kaplan–Meier analysis with the log-rank test was used to evaluate the prognostic discrimination of both pathological and model predicted Ki-67 status for DFS. Preoperative clinical variables, pathological Ki-67 status, and model predicted probabilities of high Ki-67 expression at both the 14% and 30% cut-offs (rescaled by multiplying by 10 so that hazard ratios corresponded to a 0.1-unit increase in predicted probability) were included in the univariate Cox regression analysis. Variables with substantial multicollinearity (variance inflation factors [VIFs] > 10) were excluded from the multivariable Cox regression model. Independent prognostic factors were subsequently identified using multivariable Cox regression analysis. Time-dependent ROC analysis was performed to assess the predictive accuracy of independent predictors for 5-year DFS.

### Statistical analysis

Statistical analyses were performed using R (v4.0.3), SPSS (v26.0), and MedCalc (v18.2.1). Continuous and categorical variables were compared using appropriate tests, with statistical significance defined as *P* < 0.05. Variables with *P* < 0.05 in univariate logistic regression were predefined for inclusion in the multivariable logistic regression analysis. Model performance was evaluated using ROC curve analysis, and AUCs were compared using the DeLong test. Additional metrics including accuracy, sensitivity, specificity, positive predictive value (PPV), and negative predictive value (NPV) were calculated. Clinical utility was assessed using decision curve analysis (DCA).

## Results

### Patient characteristics

A total of 636 female patients were enrolled from two independent centres. The baseline clinicopathological characteristics are summarised in Table 1. The distribution of Ki-67 expression levels was comparable across the training, internal validation, and external test cohorts (*P* > 0.05). Among all clinicopathological parameters, only lesion size exhibited a significant difference in distribution (*P* = 0.010).

**Table 1:** Clinical and radiological features of the training, internal validation, and external test cohorts

Characteristics	Training (*n* = 310)	Validation (*n* = 77)	Test (*n* = 78)	*P*
Age	50 (44, 58)	53 (45, 60)	52 (45, 59)	0.415
Size	2.47 (1.83, 3.30)	2.24 (1.60, 2.72)	2.18 (1.77, 2.66)	0.010^*^
Menopausal status				
Premenopausal	163 (52.6)	31 (40.3)	44 (56.4)	0.092
Postmenopausal	147 (47.4)	46 (59.7)	34 (43.6)	
FGT				
Fatty/scattered	50 (16.1)	13 (16.9)	20 (25.6)	0.142
Heterogeneous/extreme	260 (83.9)	64 (83.1)	58 (74.4)	
BPE				
Minimal/mild	213 (68.7)	52 (67.5)	50 (64.1)	0.738
Moderate/marked	97 (31.3)	25 (32.5)	28 (35.9)	
Presence				
Mass	238 (76.8)	56 (72.7)	66 (84.6)	0.187
Mass with NME	72 (23.2)	21 (27.3)	12 (15.4)	
Side				
Left	154 (49.7)	41 (53.2)	45 (57.7)	0.427
Right	156 (50.3)	36 (46.8)	33 (42.3)	
Focality				
Uni	268 (86.5)	65 (84.4)	64 (82.1)	0.596
Multi	42 (13.5)	12 (15.6)	14 (17.9)	
Type				
Invasive ductal cancer	276 (89.0)	72 (93.5)	70 (89.7)	0.319
Invasive lobular cancer	11 (3.6)	4 (5.2)	2 (2.6)	
Others	23 (7.4)	1 (1.3)	6 (7.7)	
Grade				
I + II	217 (70.0)	49 (63.6)	51 (65.4)	0.476
III	93 (30.0)	28 (36.4)	27 (34.6)	
ER status				
Negative	50 (16.1)	16 (20.8)	13 (16.7)	0.621
Positive	260 (83.9)	61 (79.2)	65 (83.3)	
PR status				
Negative	76 (24.5)	25 (32.5)	22 (28.2)	0.341
Positive	234 (75.5)	52 (67.5)	56 (71.8)	
HER2 status				
Negative	251 (81.0)	59 (76.6)	62 (79.5)	0.690
Positive	59 (19.0)	18 (23.4)	16 (20.5)	
Molecular subtype				
Luminal	261 (84.2)	60 (77.9)	66 (84.6)	0.393
Non-luminal	49 (15.8)	17 (22.1)	12 (15.4)	
LN status				
Negative	206 (66.5)	44 (57.1)	46 (59.0)	0.202
Positive	104 (33.5)	33 (42.9)	32 (41.0)	
Ki-67 index				
< 14%	82 (26.5)	20 (26.0)	15 (19.2)	0.415
≥ 14%	228 (73.5)	57 (74.0)	63 (80.8)	
< 30%	166 (53.5)	42 (54.5)	31 (39.7)	0.077
≥ 30%	144 (46.5)	35 (45.5)	47 (60.3)	

Data in parentheses are interquartile range for numerical variables and percentages for categorical variables. Values shown with asterisk indicate statistical significance

*FGT*, fibroglandular tissue; *BPE*, background parenchymal enhancement; *NME*, non-mass enhancement; *ER*, estrogen receptor; *PR*, progesterone receptor; *HER2*, human epidermal growth factor receptor 2; *LN*, lymph node

### Predictor identification and model development

Univariate logistic regression analysis identified tumour size as the only variable significantly associated with high Ki-67 expression at both the 14% and 30% cut-offs (both *P* < 0.001; Table 2). As no other variables met the predefined inclusion criterion, multivariable logistic regression analysis was not performed. Tumour size was subsequently used for clinical model construction. A total of 1,834 radiomics features were initially extracted from each tumour VOI. After reproducibility assessment, 1,748 features were retained. Subsequent univariate statistical filtering retained 856 and 747 features for the 14% and 30% Ki-67 cut-offs, respectively. Pearson correlation analysis was then performed to remove highly redundant features, yielding 403 and 383 candidate features for the 14% and 30% cut-offs, respectively. The mRMR algorithm was used to select the top 30 features for each cut-off, followed by LASSO regression for the final feature selection. Ultimately, 12 and 10 radiomics features were retained to construct the models for the 14% and 30% cut-offs, respectively.

**Table 2:** Univariate logistic regression analysis

Factors	Odds ratio (95%CI)	*P*
14% as the cut-off value		
Age	1.004 (0.979–1.030)	0.760
Meno	1.297 (0.779–2.157)	0.317
FGT	0.653 (0.310–1.374)	0.261
BPE	0.837 (0.489–1.432)	0.516
Presence	1.489 (0.789–2.810)	0.219
Side	1.327 (0.800-2.204)	0.273
Size	1.934 (1.450–2.579)	< 0.001^*^
Focality	0.884 (0.429–1.821)	0.738
30% as the cut-off value		
Age	0.987 (0.965–1.010)	0.255
Meno	1.038 (0.664–1.623)	0.870
FGT	0.697 (0.380–1.279)	0.244
BPE	0.831 (0.513–1.347)	0.453
Presence	1.497 (0.882–2.542)	0.135
Side	1.028 (0.658–1.607)	0.903
Size	2.665 (2.033–3.492)	< 0.001^*^
Focality	1.056 (0.550–2.025)	0.870

Values shown with asterisk indicate statistical significance

*95% CI*, 95% confidence interval; *Meno*, menopausal status; *FGT*, fibroglandular tissue; *BPE*, background parenchymal enhancement

For the radiomics models, the final classifier was selected by considering both discrimination in the internal validation cohort and consistency between the training and validation cohorts. For the 14% cut-off, ET was selected as the optimal model, showing the highest AUCs in both the training set (0.904) and internal validation set (0.793). For the 30% cut-off, XGBoost achieved the highest AUC in the training cohort (0.892) and maintained comparable performance in the validation cohort (AUC = 0.868), demonstrating robust and balanced predictive performance across datasets. Accordingly, XGBoost was selected as the final model (Supplementary Table S3 and Figure S1). Although ET and MLP achieved marginally higher AUCs in the internal validation cohort, both models showed higher AUCs in the validation cohort than in the training cohort (ET: 0.869 vs. 0.882; MLP: 0.795 vs. 0.880), an uncommon pattern that may reflect greater variability across datasets. Given its more consistent performance across cohorts, XGBoost was therefore selected for subsequent analyses.

For the deep learning models, ShuffleNet was selected as the optimal architecture for both the 14% and 30% Ki-67 cut-offs among the four evaluated candidates. It achieved the highest AUCs in the internal validation cohort for both the 14% (0.656) and 30% (0.801) cut-offs, whereas the remaining architectures yielded AUCs ranging from 0.606 to 0.631 and from 0.671 to 0.758, respectively (Supplementary Table S3).

The selected radiomics and deep learning models were subsequently evaluated in the external test cohort and, together with the clinical model, used to construct the combined models.

### Model performance evaluation

For the 14% cut-off, the radiomics model showed the highest discriminative performance among the individual models in the external test cohort, with an AUC of 0.885 (95% CI: 0.762–0.971). Its performance was significantly superior to that of the clinical model (AUC = 0.695; *P* = 0.007) and the deep learning model (AUC = 0.630; *P* = 0.013). The deep learning model showed a decrease in performance from the training cohort (AUC = 0.772) to the external test cohort (AUC = 0.630), indicating limited discriminative ability and reduced generalisability at this threshold. The combined model achieved the highest AUC of 0.895 (95% CI: 0.805–0.953) in the test cohort, significantly outperforming the clinical and deep learning models (*P* = 0.009 and *P* = 0.004, respectively), but not the radiomics model (*P* = 0.641).

For the 30% cut-off, the radiomics and deep learning models both significantly outperformed the clinical model in the external test cohort, with AUCs of 0.831, 0.825, and 0.684, respectively (*P* = 0.011 and *P* = 0.026, respectively, versus the clinical model). No significant difference was observed between the radiomics and deep learning models (*P* = 0.912). The combined model achieved the highest AUC of 0.869 (95%CI: 0.773–0.935) and significantly outperformed the clinical model (*P* < 0.001), but did not show a significant improvement over the radiomics and deep learning models (*P* = 0.232 and *P* = 0.150, respectively).

**Fig. 3:**
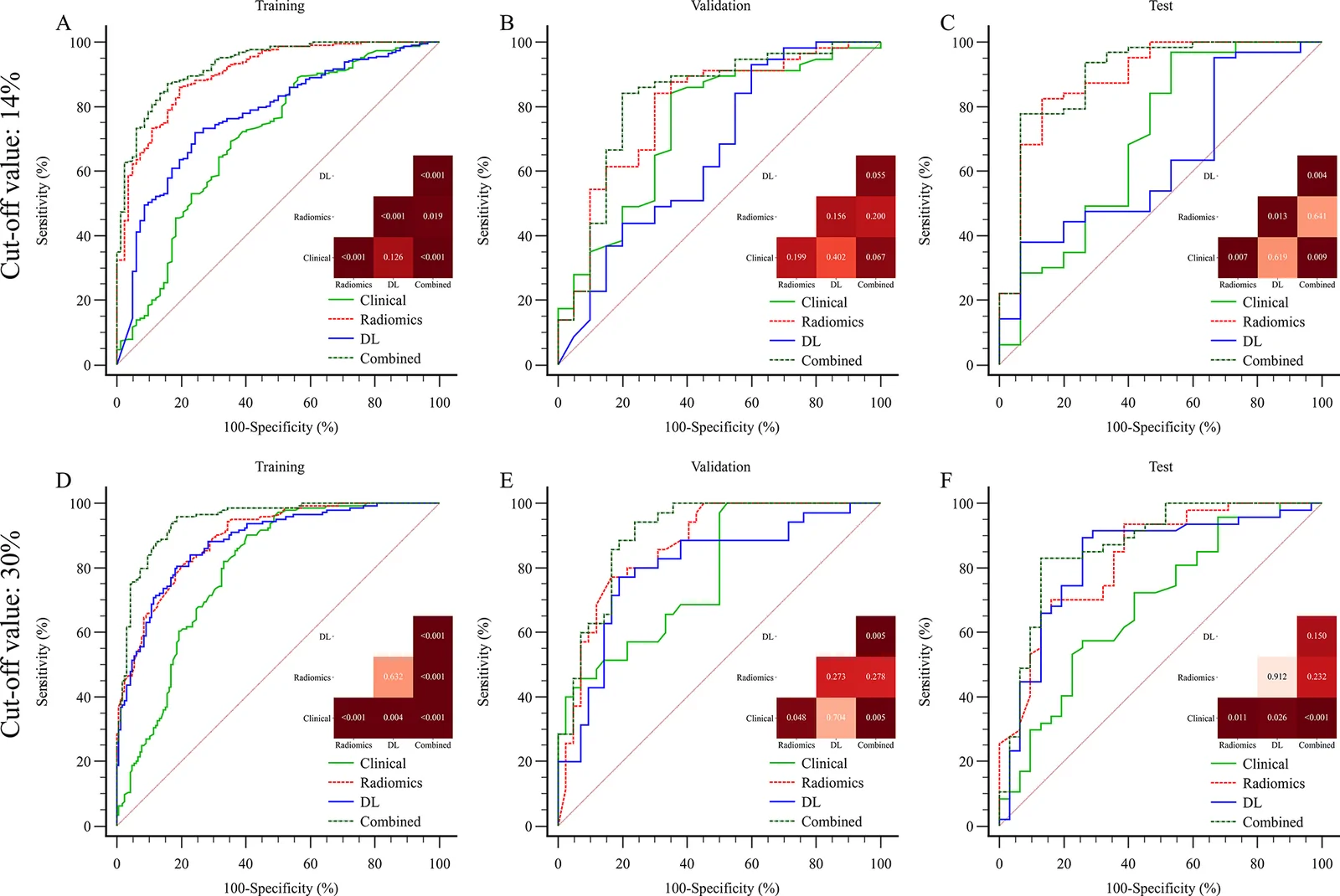
Receiver operating characteristic curves and their corresponding DeLong test *P* value heatmaps. *DL*, deep learning

**Fig. 4:**
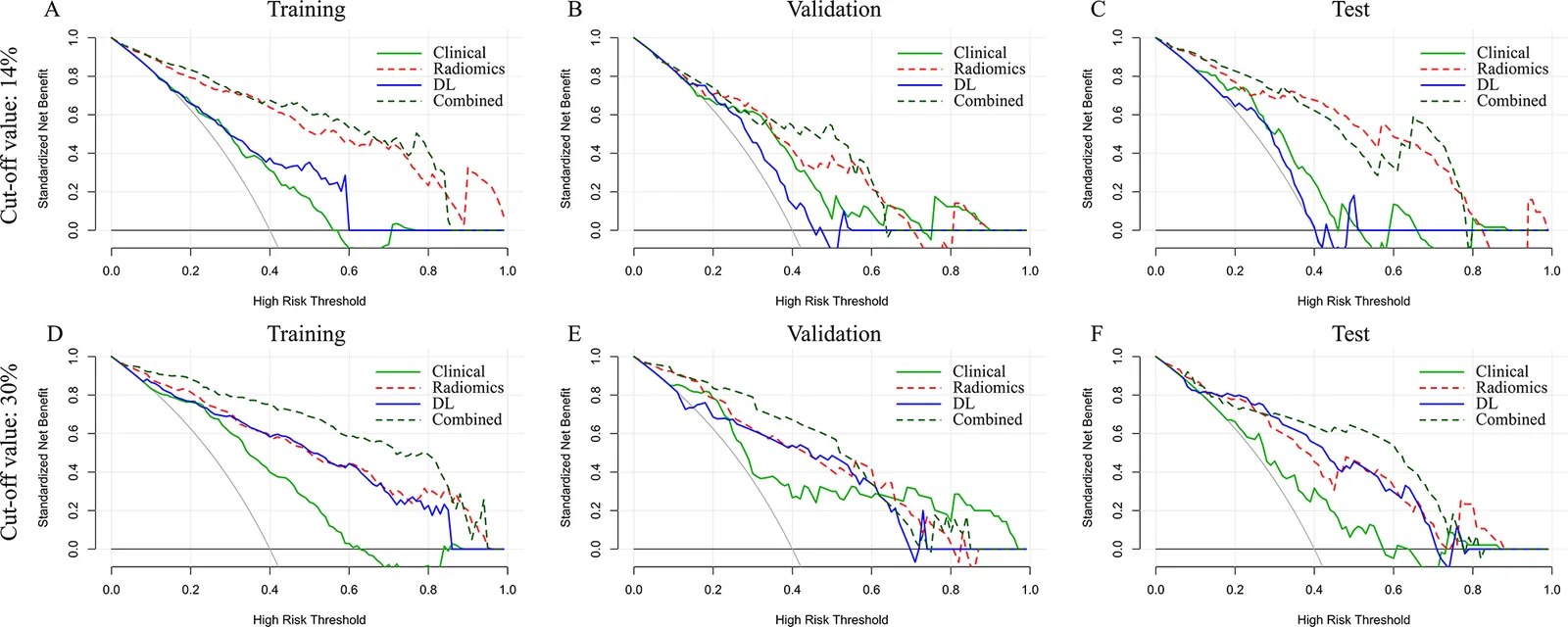
Decision curves analysis. *DL*, deep learning

Detailed metrics are shown in Table 3. ROC curves and the results of the DeLong test are presented in Fig. 3. Decision curve analysis showed the net clinical benefit of each model across a range of threshold probabilities (Fig. 4).

**Table 3:** The diagnostic performances of clinical, radiomics and deep learning models

Model	Cohort	AUC (95% CI)	ACC	Sensitivity	Specificity	PPV	NPV
14% as the cut-off value							
Clinical	Training	0.701 (0.631–0.768)	0.681	0.693	0.646	0.845	0.431
	Validation	0.741 (0.612–0.864)	0.792	0.842	0.650	0.873	0.591
	Test	0.695 (0.505–0.860)	0.872	0.968	0.467	0.884	0.778
Radiomics	Training	0.904 (0.865–0.937)	0.845	0.860	0.805	0.925	0.673
	Validation	0.793 (0.653–0.904)	0.805	0.842	0.700	0.889	0.609
	Test	0.885 (0.762–0.971)	0.833	0.825	0.867	0.963	0.542
DL	Training	0.772 (0.713–0.824)	0.729	0.719	0.756	0.891	0.492
	Validation	0.656 (0.501–0.805)	0.792	0.930	0.400	0.815	0.667
	Test	0.630 (0.475–0.783)	0.487	0.381	0.933	0.960	0.264
Combined	Training	0.929 (0.895–0.955)	0.865	0.873	0.842	0.939	0.704
	Validation	0.820 (0.716–0.898)	0.831	0.842	0.800	0.923	0.640
	Test	0.895 (0.805–0.953)	0.808	0.778	0.933	0.980	0.500
30% as the cut-off value							
Clinical	Training	0.793 (0.745–0.841)	0.739	0.903	0.596	0.660	0.876
	Validation	0.778 (0.666–0.869)	0.714	1.000	0.476	0.614	1.000
	Test	0.684 (0.562–0.804)	0.641	0.574	0.742	0.771	0.535
Radiomics	Training	0.892 (0.858–0.926)	0.803	0.813	0.795	0.775	0.830
	Validation	0.868 (0.782–0.936)	0.805	0.771	0.833	0.794	0.814
	Test	0.831 (0.740–0.918)	0.808	0.936	0.613	0.786	0.864
DL	Training	0.881 (0.842–0.916)	0.810	0.806	0.813	0.789	0.828
	Validation	0.801 (0.700-0.893)	0.792	0.771	0.810	0.771	0.810
	Test	0.825 (0.715–0.918)	0.833	0.894	0.742	0.840	0.821
Combined	Training	0.946 (0.915–0.968)	0.881	0.958	0.813	0.817	0.957
	Validation	0.903 (0.815–0.959)	0.844	0.943	0.762	0.767	0.941
	Test	0.869 (0.773–0.935)	0.846	0.830	0.871	0.907	0.771

*AUC*, area under the curve; *95% CI*, 95% confidence interval; *ACC*, accuracy; *PPV*, positive predictive value; *NPV*, negative predictive value; *DL*, deep learning

### Model interpretability using SHAP

**Fig. 5:**
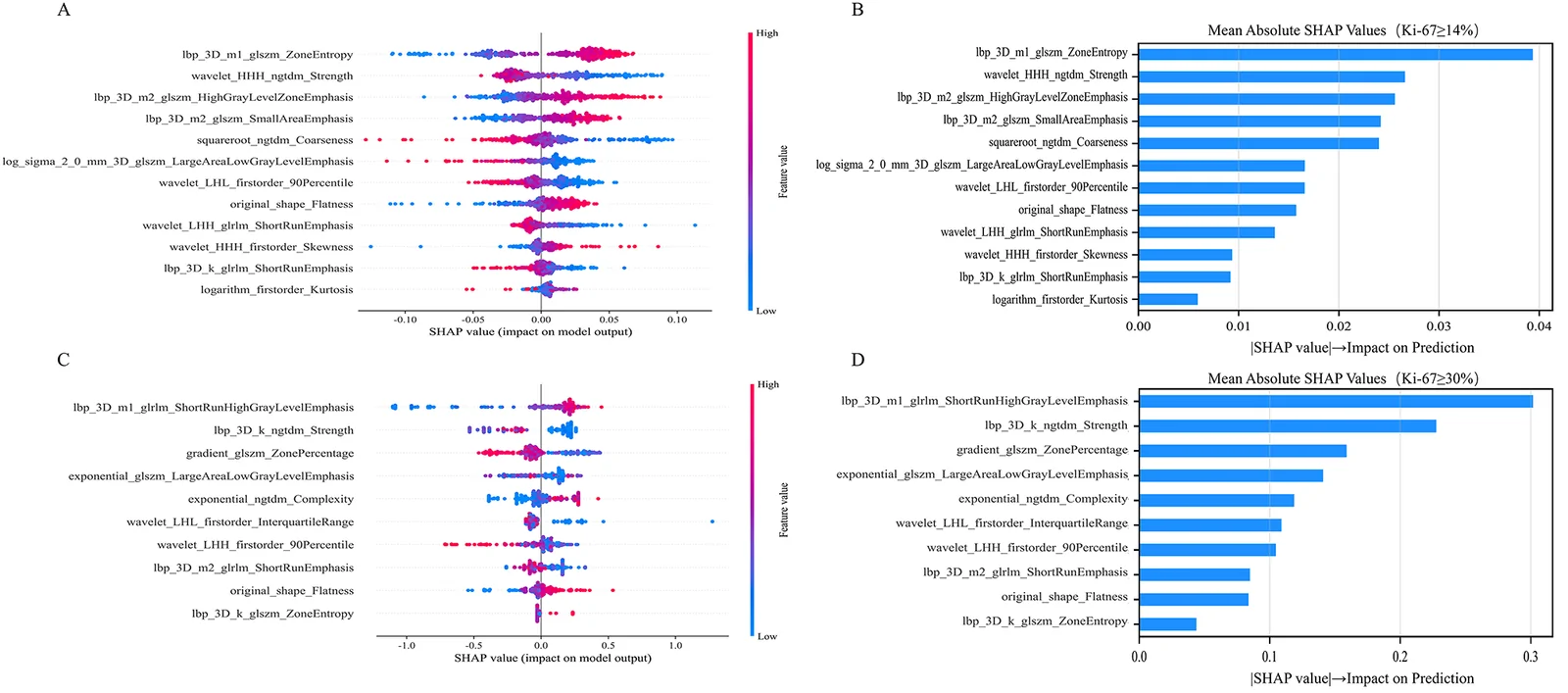
SHapley Additive exPlanation-based visualization of the radiomics model. Beeswarm and bar plots for the ExtraTree model at 14% cut-off **(A**,** B)**, and eXtreme Gradient Boosting models at 30% **(C**,** D)**

SHAP analysis revealed different feature contribution patterns for each Ki-67 threshold. Beeswarm plots (Fig. 5A, C) illustrated how individual features contributed to the model’s predictions, while summary bar plots (Fig. 5B, D) ranked these features by their overall importance. The feature lbp_3D_ml_glszm_ZoneEntropy emerged as the most important positive contributor for the 14% cut-off, whereas lbp_3D_m1_glrlm_ShortRunHighGrayLevelEmphasis was the most influential feature for the 30% cut-off.

### Survival prognostication

**Fig. 6:**
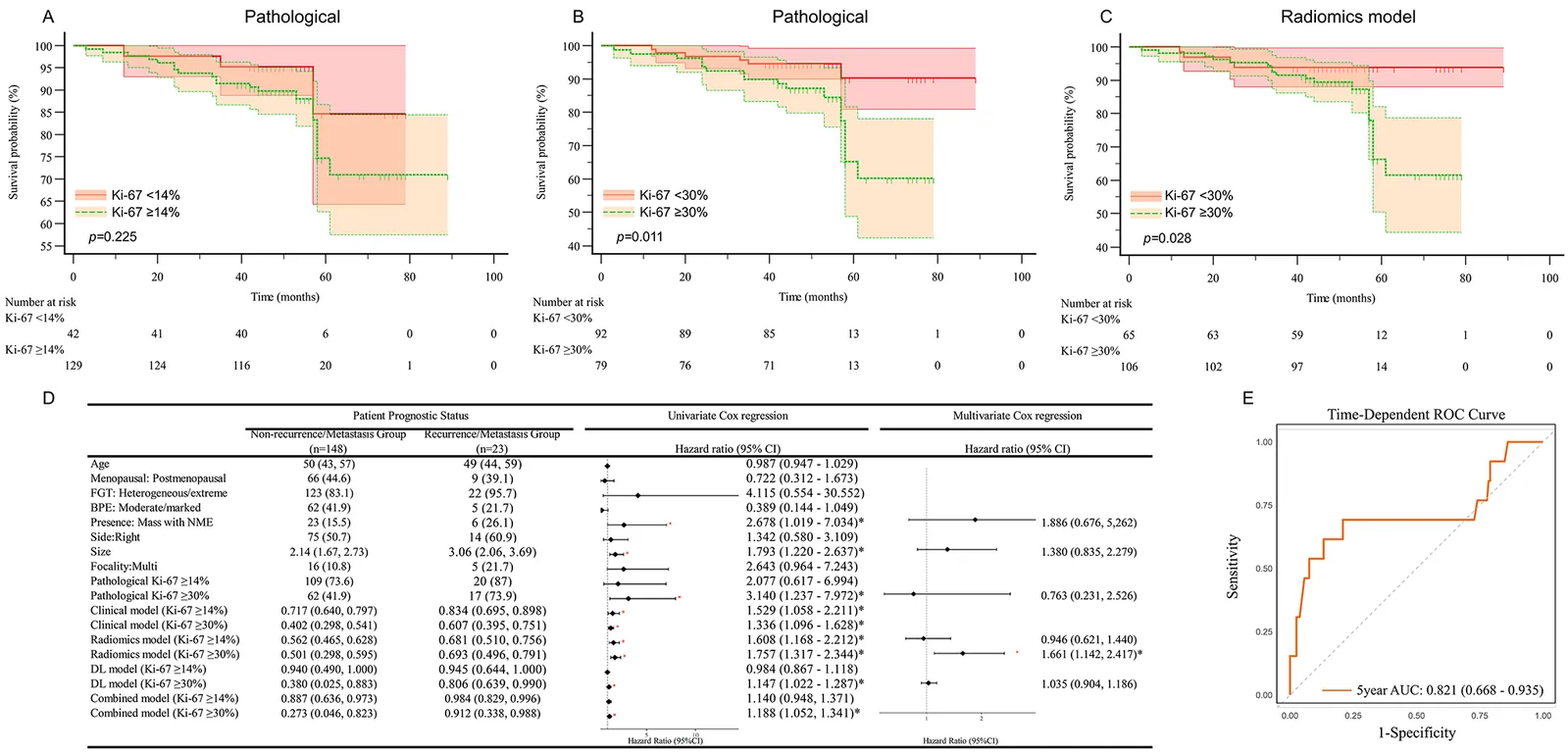
Prognostic analysis. Kaplan-Meier analysis of DFS based on Ki-67 expression: pathologically confirmed Ki-67 < 14% vs. ≥ 14% (**A**); pathologically confirmed Ki-67 < 30% vs. ≥ 30% (**B**); radiomics model-predicted Ki-67 < 30% vs. ≥ 30% (**C**); Univariate and multivariate Cox regression analysis of DFS (**D**); Time-dependent ROC curve of the radiomics score (predicting Ki-67 ≥ 30%) for 5-year DFS prediction (**E**). Values shown with asterisk indicate statistical significance. *DFS*, disease-free survival; *ROC*, receiver operating characteristic curves

In the survival analysis, Kaplan–Meier analysis showed that pathological Ki-67 ≥ 30% was associated with significantly shorter DFS compared with Ki-67 < 30% (log-rank *P* = 0.011), whereas stratification using the 14% threshold did not show a significant difference in DFS (log-rank *P* = 0.225). Univariate Cox regression identified lesion presence, size, pathological Ki-67 ≥ 30%, predicted probabilities of Ki-67 ≥ 14% from the clinical and radiomics models, and predicted probabilities of Ki-67 ≥ 30% from all models as significantly associated with DFS (all *P* < 0.05). Due to substantial multicollinearity (VIFs > 10) among tumour size, the clinical model, and the combined model, only tumour size was retained for the multivariable model given its greater clinical availability. In the multivariable Cox regression analysis, the radiomics score for predicting Ki-67 ≥ 30% remained an independent predictor of worse DFS (HR = 1.661, 95% CI: 1.142–2.417; *P* = 0.008). Patients classified as having high Ki-67 status (≥ 30%) by the radiomics model had significantly shorter DFS than those classified as low Ki-67 status (log-rank *P* = 0.028). The radiomics score also showed favourable predictive accuracy for 5-year DFS, with an AUC of 0.821. The prognostic analysis results are presented in Fig. 6.

## Discussion

This study developed clinical and CE-CBBCT-based models for the preoperative, non-invasive prediction of Ki-67 expression status at different cut-off values. Among these models, the radiomics model demonstrated consistently robust performance and outperformed both the clinical and deep learning models in the external test cohort across different cut-off values. Although the combined model achieved the highest AUCs, its performance improvement over the radiomics model was not statistically significant in the test cohort. Furthermore, the radiomics score for predicting Ki-67 ≥ 30% demonstrated prognostic value for DFS. These findings suggest that CE-CBBCT-based radiomics represents a promising non-invasive approach for predicting Ki-67 expression status, with potential applications in preoperative prognostic assessment and clinical decision-making.

Preoperative non-invasive prediction of Ki-67 expression has been widely investigated using various imaging modalities, particularly ultrasound and MRI. For instance, Wu et al. [12] developed an interpretable machine learning model based on intratumoural and peritumoural ultrasound radiomics features, demonstrating the potential of ultrasound in predicting Ki-67 status. Similarly, several MRI-based studies have achieved promising results [13, 14, 29]. Kayadibi et al. [14] developed an MRI-based model in 159 patients, reporting AUCs of 0.785 and 0.744 at the 14% and 20% thresholds, respectively. However, their study did not evaluate the 30% threshold recommended by the International Ki-67 Working Group [2] and lacked external validation. In contrast, our study contributes to this field by introducing a radiomics framework based on the emerging technique of CE-CBBCT. Our CE-CBBCT-based model demonstrated competitive performance, with AUCs of 0.885 and 0.831 for the 14% and 30% cut-offs, respectively, in the external test cohort. These results are comparable to those reported in previous MRI- and ultrasound-based studies. By simultaneously evaluating two clinically relevant thresholds and employing an external test cohort, our study addresses some limitations of previous studies and supports the potential clinical utility of CE-CBBCT-based radiomics for Ki-67 prediction.

Our research identified tumour size as the sole independent predictor of high Ki-67 expression at both 14% and 30% cut-offs, consistent with Wang et al. [30] but contrasting with Liu et al. [13] and Zhang et al. [29]. Despite its statistical significance, the low AUC of tumour size underscores its limited discriminatory power, highlighting the need for more comprehensive imaging-based predictive models.

Both radiomics and deep learning have emerged as promising tools for predicting Ki-67 status in various malignancies [31, 32], providing quantitative imaging information beyond conventional clinical variables. Although deep learning excels in automated feature extraction and end-to-end training [33], our radiomics model demonstrated superior overall performance, consistent with previous studies [34, 35]. One possible explanation is the limited sample size and class imbalance, which can impair model generalisability. At the 14% cut-off (high: low ≈ 3:1), the deep learning model showed limited generalisation. In contrast, under the balanced 30% cut-off (ratio ≈ 1:1), it performed comparably to the radiomics model. These results suggest that the potential advantages of deep learning may not be fully realized in limited and imbalanced medical imaging datasets, as high-capacity neural networks generally require larger and more balanced cohorts to learn stable representations [36]. To further assess the influence of network architecture, we compared four CNN models. Although ResNet50, ResNet101, and DenseNet121 are widely used and have demonstrated excellent performance in medical imaging tasks [37], none outperformed ShuffleNet in our dataset, highlighting the importance of selecting model architectures according to specific dataset characteristics. Future improvements may include expanding multi-centre datasets, improving class balance, optimizing 3D network architectures, incorporating transfer learning or self-supervised pretraining, and exploring advanced vision transformer-based models.

Radiomics may be relatively more robust in such settings because it uses predefined quantitative features followed by feature selection [38]. Nevertheless, sample size and class distribution can also influence radiomics model performance. At the 14% cut-off, the ET classifier demonstrated signs of potential overfitting. This may reflect the inherent complexity of ET as an ensemble tree-based model, which can capture complex nonlinear feature interactions in the training cohort, while showing reduced stability in an independent validation cohort. The relatively small validation cohort and class imbalance at this threshold may have further contributed to this finding.

The combined model achieved the highest AUCs among all evaluated models; however, its improvement over the radiomics model was not statistically significant in the test cohorts. This finding suggests that CE-CBBCT-based radiomics features may already capture much of the imaging information related to Ki-67 expression, limiting the incremental value of adding deep learning and clinical information. Further studies in larger and more diverse cohorts are needed to clarify the value of multimodal model integration.

Beyond predictive accuracy, model interpretability is crucial for clinical translation. To this end, we applied SHAP analysis to decipher the decision logic of our radiomics model. SHAP analysis identified lbp_3D_ml_glszm_ZoneEntropy and lbp_3D_m1_glrlm_ShortRunHighGrayLevelEmphasis as the most influential features for the 14% and 30% Ki-67 thresholds, respectively. glszm_ZoneEntropy quantifies the randomness and complexity of the gray-level size-zone distribution, with higher values indicating more heterogeneous spatial distributions of homogeneous gray-level zones. glszm_ZoneEntropy has been reported to correlate strongly with features reflecting intra-VOI inhomogeneity, elevated voxel intensity, and high tumour heterogeneity [39]. glrlm_ShortRunHighGrayLevelEmphasis emphasizes short runs with high gray-level values, reflecting fine-scale, high-intensity textural patterns. In our study, both features were extracted after 3D local binary pattern transformation, meaning that they describe local textural heterogeneity on transformed images rather than direct intensity patterns on the original CE-CBBCT images [40]. Therefore, their biological interpretation should be made cautiously. These features may indirectly reflect intratumoural heterogeneity and heterogeneous enhancement. Further radiology-pathology correlation studies are needed to clarify their precise histopathological correlates [41]. The SHAP analysis also indicated that the most influential features differed between the two thresholds. This result may be mainly attributable to the distinct classification tasks defined by the two cut-offs. Changing the threshold alters the composition of the positive and negative groups, as well as the distribution of tumour aggressiveness within each group, which may consequently shift the optimal radiomics features used for discrimination. In addition, different optimal classifiers were selected for the 14% and 30% thresholds, which may also contribute to the variation in SHAP feature weights.

In addition to textural features, the morphological feature “original_shape_flatness” was also associated with Ki-67 status, which is calculated as the ratio of the shortest to the longest axis. Values approaching 1 indicate spheroidal morphology, whereas lower values suggest flattening. This feature, previously associated with tumour aggressiveness in renal malignancies [42], correlated with higher Ki-67 expression likelihood in our study. We hypothesize that spheroidal tumour morphology may reflect more coordinated cell proliferation with balanced growth vectors, in contrast to the disorganized expansion suggested by flattened shapes. This interpretation is supported by molecular studies showing that Ki-67-active tumours often overexpress matrix metalloproteinases [43], which could reduce stromal resistance and potentially facilitate more symmetrical tumour growth.

Ki-67 is an established prognostic marker, with higher expression levels often correlating with a poorer prognosis [2]. While pathologically high Ki-67 (> 30%) correlated with shorter DFS in our research, it was not an independent prognostic factor in multivariate analysis. In contrast, the radiomics score for the 30% cut-off appeared to retain independent prognostic value for DFS. This may be attributable to the model’s capacity to capture tumour heterogeneity and microenvironmental features, biological traits linked to aggressiveness and treatment resistance [44], which may not always be fully captured by sampling-limited pathological assessments [45]. Thus, the exploratory findings suggest that the radiomics model may have the potential to provide preoperative prognostic information and help identify patients at higher risk of recurrence, supporting individualized clinical decision-making.

Our study has several limitations. First, as CBBCT remains an emerging breast imaging modality, the sample size was relatively limited, and class imbalance was present, particularly at the 14% Ki-67 cut-off. Although class imbalance was partly addressed by two-centre data collection and SMOTE, the limited and imbalanced dataset may have affected model robustness and generalisability. The prognostic analysis should therefore be considered preliminary and requires validation in larger prospective cohorts with longer follow-up and sufficient outcome events. Second, the retrospective design may have introduced selection bias. The long recruitment period may also have contributed to temporal heterogeneity related to changes in clinical practice and pathological assessment. Third, NME, a breast cancer subtype with distinct imaging features [46], was excluded due to challenges in delineating boundaries. This may limit model applicability to mass-forming cancers. Future studies with NME cases are needed to validate broader model applicability. Fourth, although standardized CE-CBBCT protocols were used at both centres in this study, future multicentre studies with larger and more heterogeneous datasets should incorporate harmonization strategies to minimize inter-centre variability and further improve model generalisation [47]. Fifth, tumour VOIs were manually delineated, which may have introduced operator-dependent variability and limited efficiency. Although inter-observer reproducibility was assessed, intra-observer reproducibility was not evaluated. Future studies should include both inter- and intra-observer analyses and explore automated or semi-automated segmentation to reduce operator-related subjectivity [48]. Finally, the deep learning model used a VOI derived from the minimum bounding cube of the tumour segmentation, which provided standardized volumetric input and limited local contextual information. However, the contribution of peritumoural tissue was not specifically investigated. Given the emerging evidence supporting the prognostic and predictive value of the peritumoural microenvironment [49], future studies should integrate both intratumoural and peritumoural information and optimize peritumoural margin settings to better characterize tumour heterogeneity.

## Conclusion

The radiomics model based on CE-CBBCT demonstrated robust performance in the preoperative prediction of Ki-67 expression in breast cancer patients and may serve as a valuable adjunct to CNB. Furthermore, it provides a novel imaging biomarker with preliminary prognostic value for DFS, suggesting its potential to enhance personalized management of breast cancer patients. Further validation in larger, independent cohorts is required to confirm its generalizability.

## Supplementary Information

Below is the link to the electronic supplementary material.

Supplementary Material 1

## Data Availability

The datasets generated and/or analyzed during the current study are not publicly available due to data privacy and intellectual property issues but are available from the corresponding author on reasonable request.

## Abbreviations

AdaBoost: Adaptive Boosting
AUC: Area under the curve
BPE: Background parenchymal enhancement
95% CI: 95% confidence interval
CE-CBBCT: Contrast-enhanced cone-beam breast CT
CNB: Core needle biopsy
DFS: Disease-free survival
DL: Deep learning
DCA: Decision curve analysis
ER: Estrogen receptor
ET: ExtraTrees
FGT: Fibroglandular tissue
HER2: Human epidermal growth factor receptor-2
HR: Hazard ratio
ICC: Intraclass correlation coefficient
IHC: Immunohistochemistry
IKWG: International Ki-67 in Breast Cancer Working Group
LASSO: Least absolute shrinkage and selection operator
LN: Lymph node
LR: Logistic regression
MLP: Multi-Layer Perceptron
MS: Molecular subtype
mRMR: Minimum redundancy maximum relevance
NME: Non-mass enhancement
OR: Odds ratios
PR: Progesterone receptor
PPV: Positive predictive value
NPV: Negative predictive value
RF: Random forest
ROC: Receiver operating characteristic
SGD: Stochastic Gradient Descent
SHAP: SHapley Additive exPlanation
SMOTE: Synthetic minority oversampling technique
VOIs: Volumes of interest
XGBoost: eXtreme Gradient Boosting
3D: Three dimensional
