# Supplementary material for: Preoperative prediction of Ki-67 expression and prognosis in breast cancer based on contrast-enhanced cone-beam breast CT: a two-centre study
Source: Cancer Imaging. 2026 Jun 26;26:128. doi: 10.1186/s40644-026-01082-0 (PMC13576104; doi:10.1186/s40644-026-01082-0)
Supplement: Supplementary file 1 [file 40644_2026_1082_MOESM1_ESM.docx]

**SUPPLEMENTARY MATERIALS**

**Software and Programming Environment**

All radiomics preprocessing, feature selection, machine learning modelling, data balancing, and model interpretation were performed in Python version 3.7.12. Radiomics feature extraction was conducted using PyRadiomics version 3.0.1. The machine learning classifiers, including Random Forest, ExtraTrees, AdaBoost, and Multi-Layer Perceptron, were implemented using scikit-learn version 1.0.2. XGBoost was implemented using the xgboost package version 1.5.2. SMOTE was performed using the imbalanced-learn package version 0.9.0. SHAP analysis was conducted using the shap package version 0.41.0. The 3D deep learning models were implemented using PyTorch version 1.11.0 within the Onekey AI platform. Data processing and numerical analyses were additionally supported by NumPy version 1.21.6, pandas version 1.3.5, and SciPy version 1.7.3.

**Table S1** Hyperparameter search space and selected parameters for radiomics models

| **Classifier** | **Hyperparameters searched and Search space** | **Final selected parameters (14% cut-off)** | **Final selected parameters (30% cut-off)** |
| --- | --- | --- | --- |
| Random Forest | n_estimators: [50, 100, 200] | n_estimators=50 | n_estimators=50 |
|  | max_depth: [3, 4, 5, 6, 7] | max_depth=3 | max_depth=5 |
|  | min_samples_split: [2, 3, 4, 5] | min_samples_split=4 | min_samples_split=4 |
| ExtraTrees | n_estimators: [50, 60, 100, 200] | n_estimators=60 | n_estimators=50 |
|  | max_depth: [3, 4, 5, 6] | max_depth=5 | max_depth=5 |
|  | min_samples_split: [2, 3, 4] | min_samples_split=2 | min_samples_split=2 |
| XGBoost | n_estimators: [50, 100, 200] | n_estimators=100 | n_estimators=100 |
|  | max_depth: [2, 3, 4, 5] | max_depth=3 | max_depth=3 |
|  | min_child_weight: [0.1, 0.2, 0.5, 1] | min_child_weight=0.1 | min_child_weight=0.2 |
| AdaBoost | n_estimators: [10, 50, 100] | n_estimators=50 | n_estimators=50 |
|  | learning_rate: [0.01, 0.1, 0.5, 1.0] | learning_rate= 0.01 | learning_rate= 0.01 |
| MLP | hidden_layer_sizes: [(64,), (128,), (64, 32), (128, 64)] | hidden_layer_sizes=(64, 32) | hidden_layer_sizes=(64, 32) |
|  | activation: ['relu', 'tanh', 'logistic'] | activation='relu' | activation='relu' |
|  | solver: ['adam', 'sgd', 'lbfgs'] | solver='adam' | solver='adam' |
|  | alpha: [0.0001, 0.001, 0.01, 0.1] | alpha=0.001 | alpha=0.001 |
|  | max_iter: [100, 200, 500] | max_iter=200 | max_iter=200 |

*XGBoost*, eXtreme Gradient Boosting; *AdaBoost*, Adaptive Boosting; *MLP*, Multi-Layer Perceptron

**Table S2** Training settings for deep learning models

| **Item** | **Setting** |
| --- | --- |
| Architectures | 3D ShuffleNet, 3D ResNet50, 3D ResNet101, 3D DenseNet121 |
| Framework | PyTorch version 1.11.0 |
| Input size | 64, 64, 48 |
| Batch size | 4 |
| Optimizer | Stochastic Gradient Descent |
| Initial learning rate | 0.001 |
| Momentum | 0.9 |
| Weight decay | 1 × 10^-4^ |
| Loss function | Binary Cross-Entropy |
| Epochs | 80 |
| Learning rate scheduler | ReduceLROnPlateau |
| Data augmentation | X-, Y-, and Z-axis flipping |
| Model selection criterion | lowest validation loss |

*3D*, three-dimensional

**Table S3** Performance of multiple radiomics and deep learning models

|  | **Model** | **Cohort** | **AUC (95% CI)** | **ACC** | **Sensitivity** | **Specificity** | **PPV** | **NPV** |
| --- | --- | --- | --- | --- | --- | --- | --- | --- |
| 14% as the cut-off value: | | | | | | | | |
| Radiomics models | | | | | | | | |
| RF | | Training | 0.839 (0.793-0.884) | 0.742 | 0.693 | 0.878 | 0.940 | 0.507 |
|  |  | Validation | 0.792 (0.670-0.914) | 0.779 | 0.789 | 0.750 | 0.900 | 0.556 |
| **ET** | | **Training** | **0.904 (0.865-0.937)** | **0.845** | **0.860** | **0.805** | **0.925** | **0.673** |
|  |  | **Validation** | **0.793 (0.653-0.904)** | **0.805** | **0.842** | **0.700** | **0.889** | **0.609** |
| XGBoost | | Training | 0.897 (0.862-0.931) | 0.752 | 0.680 | 0.951 | 0.975 | 0.517 |
|  |  | Validation | 0.756 (0.645-0.866) | 0.701 | 0.702 | 0.700 | 0.870 | 0.452 |
| AdaBoost | | Training | 0.838 (0.791-0.884) | 0.745 | 0.728 | 0.793 | 0.907 | 0.512 |
|  |  | Validation | 0.747 (0.637-0.857) | 0.649 | 0.579 | 0.850 | 0.917 | 0.415 |
| MLP | | Training | 0.865 (0.821-0.909) | 0.816 | 0.842 | 0.744 | 0.901 | 0.629 |
|  |  | Validation | 0.768 (0.634-0.903) | 0.818 | 0.860 | 0.700 | 0.891 | 0.636 |
| Deep learning models | | | | | | | | |
| **ShuffleNet** | | **Training** | **0.772 (0.713-0.824)** | **0.729** | **0.719** | **0.756** | **0.891** | **0.492** |
|  |  | **Validation** | **0.656 (0.501-0.805)** | **0.792** | **0.930** | **0.400** | **0.815** | **0.667** |
| Resnet50 | | Training | 0.649 (0.575-0.728) | 0.720 | 0.794 | 0.513 | 0.820 | 0.472 |
|  |  | Validation | 0.612 (0.459-0.760) | 0.702 | 0.878 | 0.200 | 0.758 | 0.364 |
| Resnet101 | | Training | 0.653 (0.583-0.718) | 0.649 | 0.663 | 0.610 | 0.826 | 0.394 |
|  |  | Validation | 0.606 (0.437-0.747) | 0.650 | 0.755 | 0.350 | 0.768 | 0.334 |
| DenseNet121 | | Training | 0.758 (0.700-0.813) | 0.713 | 0.724 | 0.683 | 0.864 | 0.471 |
|  |  | Validation | 0.631 (0.478-0.773) | 0.741 | 0.930 | 0.200 | 0.769 | 0.500 |
| 30% as the cut-off value: | | | | | | | | |
| Radiomics models | | | | | | | | |
| RF | | Training | 0.834 (0.790-0.878) | 0.752 | 0.701 | 0.795 | 0.748 | 0.754 |
|  |  | Validation | 0.778 (0.676-0.880) | 0.675 | 0.800 | 0.571 | 0.609 | 0.774 |
| ET | | Training | 0.869 (0.832-0.907) | 0.790 | 0.708 | 0.861 | 0.816 | 0.773 |
|  |  | Validation | 0.882 (0.804-0.961) | 0.831 | 0.829 | 0.833 | 0.806 | 0.854 |
| **XGBoost** | | **Training** | **0.892 (0.858-0.926)** | **0.803** | **0.813** | **0.795** | **0.775** | **0.830** |
|  |  | **Validation** | **0.868 (0.782-0.936)** | **0.805** | **0.771** | **0.833** | **0.794** | **0.814** |
| AdaBoost | | Training | 0.846 (0.804-0.888) | 0.745 | 0.618 | 0.855 | 0.788 | 0.721 |
|  |  | Validation | 0.826 (0.736-0.916) | 0.766 | 0.771 | 0.762 | 0.730 | 0.800 |
| MLP | | Training | 0.795 (0.746-0.843) | 0.723 | 0.556 | 0.867 | 0.784 | 0.692 |
|  |  | Validation | 0.880 (0.805-0.956) | 0.805 | 0.914 | 0.714 | 0.727 | 0.909 |
| Deep learning models | | | | | | | | |
| **ShuffleNet** | | **Training** | **0.881 (0.842-0.916)** | **0.810** | **0.806** | **0.813** | **0.789** | **0.828** |
|  |  | **Validation** | **0.801 (0.700-0.893)** | **0.792** | **0.771** | **0.810** | **0.771** | **0.810** |
| Resnet50 | | Training | 0.688 (0.633-0.747) | 0.659 | 0.632 | 0.681 | 0.632 | 0.681 |
|  |  | Validation | 0.681 (0.562-0.793) | 0.689 | 0.886 | 0.524 | 0.608 | 0.847 |
| Resnet101 | | Training | 0.787 (0.734-0.833) | 0.746 | 0.737 | 0.754 | 0.722 | 0.767 |
|  |  | Validation | 0.671 (0.549-0.784) | 0.637 | 0.543 | 0.715 | 0.613 | 0.653 |
| DenseNet121 | | Training | 0.859 (0.818-0.897) | 0.788 | 0.771 | 0.802 | 0.771 | 0.802 |
|  |  | Validation | 0.758 (0.640-0.867) | 0.715 | 0.858 | 0.596 | 0.639 | 0.834 |

Models highlighted in bold were selected for subsequent analyses.

*AUC*, area under the curve; *95% CI*, 95% confidence interval;ACC, accuracy; *PPV,* positive predictive value; *NPV,* negative predictive value; *RF,* random forest; *ET,* ExtraTrees; XGBoost, eXtreme Gradient Boosting; *AdaBoost,* Adaptive Boosting; *MLP,* Multi-Layer Perceptron

**Figure S1** The heatmap of the DeLong test results of five machine learning radiomics models

*XGBoost,* eXtreme Gradient Boosting; *AdaBoost*, Adaptive Boosting; *MLP*, Multi-Layer Perceptron; *RF*, Random Forest; *ET*, ExtraTrees
